# Exercise Stress Test Late after Arrhythmic versus Nonarrhythmic Presentation of Myocarditis

**DOI:** 10.3390/jpm12101702

**Published:** 2022-10-12

**Authors:** Giovanni Peretto, Simone Gulletta, Massimo Slavich, Corrado Campochiaro, Davide Vignale, Giacomo De Luca, Anna Palmisano, Andrea Villatore, Stefania Rizzo, Giulio Cavalli, Monica De Gaspari, Elena Busnardo, Luigi Gianolli, Lorenzo Dagna, Cristina Basso, Antonio Esposito, Simone Sala, Paolo Della Bella, Patrizio Mazzone

**Affiliations:** 1 Department of Cardiac Electrophysiology and Arrhythmology, IRCCS San Raffaele Scientific Institute, 20132 Milan, Italy; 2 Myocarditis Disease Unit, IRCCS San Raffaele Scientific Institute, 20132 Milan, Italy; 3 School of Medicine, Vita-Salute San Raffaele University, 20132 Milan, Italy; 4 Department of Cardiology, IRCCS San Raffaele Scientific Institute, 20132 Milan, Italy; 5 Unit of Immunology, Rheumatology, Allergy and Rare Diseases (UnIRAR), IRCCS San Raffaele Scientific Institute, 20132 Milan, Italy; 6 Experimental Imaging Center, Radiology Unit, IRCCS San Raffaele Scientific Institute, 20132 Milan, Italy; 7 Department of Cardiac Thoracic Vascular Sciences and Public Health, Cardiovascular Pathology, Padua University, 35128 Padua, Italy; 8 Nuclear Medicine Unit, IRCCS San Raffaele Scientific Institute, 20132 Milan, Italy

**Keywords:** exercise stress test, physical activity, myocarditis, ventricular arrhythmia, cardiac magnetic resonance, endomyocardial biopsy

## Abstract

***Background***. Exercise stress test (EST) has been scarcely investigated in patients with arrhythmic myocarditis. ***Objectives***. To report the results of EST late after myocarditis with arrhythmic vs. nonarrhythmic presentation. ***Methods***. We enrolled consecutive adult patients with EST performed at least six months after acute myocarditis was diagnosed using gold-standard techniques. Patients with ventricular arrhythmia (VA) at presentation were compared with the nonarrhythmic group. Adverse events occurring during follow-up after EST included cardiac death, disease-related rehospitalization, malignant VA, and proven active myocarditis. ***Results***. The study cohort was composed of 128 patients (age 41 ± 9 y, 70% males) undergoing EST after myocarditis. Of them, 64 (50%) had arrhythmic presentation. EST was performed after 15 ± 4 months from initial diagnosis, and was conducted on betablockers in 75 cases (59%). During EST, VA were more common in the arrhythmic group (43 vs. 4, *p* < 0.001), whereas signs and symptoms of ischemia were more prevalent in the nonarrhythmic one (6 vs. 1, *p* = 0.115). By 58-month mean follow-up, 52 patients (41%) experienced adverse events, with a greater prevalence among arrhythmic patients (39 vs. 13, *p* < 0.001). As documented both in the arrhythmic and nonarrhythmic subgroups, patients had greater prevalence of adverse events following a positive EST (40/54 vs. 12/74 with negative EST, *p* < 0.001). Electrocardiographic features of VA during EST correlated with the subsequent inflammatory restaging of myocarditis. Nonarrhythmic patients with uneventful EST both on- and off-treatment were free from subsequent adverse events. ***Conclusions***. Late after the arrhythmic presentation of myocarditis, EST was frequently associated with recurrent VA. In both arrhythmic and nonarrhythmic myocarditis, EST abnormalities correlated with subsequent adverse outcomes.

## 1. Introduction

Exercise stress test (EST) is an informative diagnostic tool allowing assessment of both inducible myocardial ischemia and arrhythmia [1]. To date, however, EST is under-investigated in myocarditis, an inflammatory disease of the myocardium initiated by viral infections, toxic agents, or dysimmune processes and frequently affecting young and otherwise healthy subjects [2]. In fact, due to the potentially harmful effects of strenuous physical activity during the active inflammatory stage of the disease [3], EST is currently contraindicated in patients diagnosed with acute myocarditis [2,4]. On the other hand, EST is considered a safe and informative technique after myocarditis healing [2,4]. Consistently, international guideline documents agree in recommending the use of EST after six months from acute myocarditis before resuming competitive sport participation [4,5]. In this setting, the absence of arrhythmia and ischemia signs is needed to demonstrate safety during incremental physical activity [6]. Remarkably, the evidence currently supporting the role of EST in myocarditis focuses on the classic acute coronary syndrome (ACS)-like presentation of myocarditis, which is the most common and benign scenario [7,8,9]. Currently, there is a lack of consistent data about EST in the remaining clinical presentations, in particular in patients with ventricular arrhythmia (VA) at the time of diagnosis. The issue is demanding since VA associated with myocarditis may be life-threatening and have been described both during the acute and chronic phases of the disease [10].

The aim of our study is to compare the results of EST at late stage of arrhythmic vs. nonarrhythmic presentation of myocarditis.

## 2. Materials and Methods

### 2.1. Study Design

This study, observational and prospective, was performed at a referral center for the management of arrhythmic myocarditis. Following the local institutional review board approval, written informed consent was obtained from all participants (MYOCAR, 24/01/2018). From January 2013 to January 2021, we enrolled consecutive inhospital patients with myocarditis, undergoing EST late after clinical presentation. In detail, inclusion criteria were: (1) age ≥ 18 years; (2) acute clinical presentation with myocarditis diagnosed by gold standard techniques, namely cardiac magnetic resonance (CMR) and/or endomyocardial biopsy (EMB); (3) EST performed at least six months after myocarditis diagnosis.

Patients presenting with VA constituted the study group and were compared with controls without VA. For the purposes of the study, VA included ventricular fibrillation (VF), either sustained or nonsustained ventricular tachycardia (VT; NSVT), or ventricular ectopies (VE) of Lown’s grade ≥2 [11].

Exclusion criteria were: catheter ablation of VT performed before EST; EST not performed or contraindicated due to clinical instability; and loss to follow-up.

### 2.2. Myocarditis Diagnosis

EMB-proven active myocarditis was defined, as recommended [2], based on histological (inflammatory infiltrates and myocyte degeneration fulfilling the Dallas criteria), immunohistochemical (≥14 leucocytes/mm^2^ and CD3+ T-lymphocytes ≥7 cells/mm^2^), and molecular criteria (polymerase chain reaction, to identify or exclude viral etiology). EMB was performed by percutaneous right ventricular sampling under fluoroscopic and echocardiographic guidance [12]. CMR-proven myocarditis was defined by the standard and updated Lake Louise criteria (LLC) [13,14] in patients enrolled before and after 2016, respectively. In detail, CMR was performed on a 1.5 T scanner (Achieva dStream; Philips Medical Systems, Eindhoven, The Netherlands) equipped with a 32-channel phased-array coil. Myocardial edema was evaluated using black blood T2 short-tau inversion recovery (STIR) images. Modified Look-Locker inversion recovery sequences and gradient-(echo planar imaging) and spin-echo multi-echo sequences were used for T1 mapping and T2 mapping, respectively. Late gadolinium enhancement (LGE) images were acquired 10 min after gadolinium injection using 2D T1 weighted segmented inversion-recovery gradient-echo sequences, and analyzed on two orthogonal planes. The correct inversion time was determined using the Look-Locker technique. Extracellular volume (ECV) was obtained according to recommended standards [15].

### 2.3. Treatment and Follow-Up

Treatment for myocarditis was patient-tailored, including optimal medical treatment and cardiac device implant, based on international guideline recommendations [16,17] and the experience of the center [18]. Upon clinical indication, immunosuppressive therapy (IST) was applied to patients with virus-negative myocarditis [19]. In particular, IST use was driven by persistent symptomatic troponin release, left ventricular systolic dysfunction, and sustained or recurrent VA. For all patients, regular follow-up at a dedicated outpatient multidisciplinary facility [20] was obtained every three months by multimodal reassessment (blood exams including cardiac biomarkers, transthoracic echocardiogram, and 12-lead 24-h Holter ECG). To allow myocarditis restaging, either CMR or EMB was repeated during follow-up based on patient symptoms and clinical reassessment. In patients with implanted cardiac devices or contraindications to CMR, 18F-Fluorodeoxyglucose positron emission tomography (FDG-PET) scan was obtained instead [21].

### 2.4. EST

As per protocol and routine clinical practice, EST was performed at least six months after diagnosis of acute myocarditis. Judgement of clinical stability, including normalization of T-troponin, absence of episodes of sustained VT or VF over the last six months, and no worsening in left ventricular ejection fraction (LVEF), as compared to baseline assessment, was obtained before indicating EST. For patients receiving IST, treatment termination was an additional required condition.

EST was conducted in designed labs by personnel blinded to the study purposes, in compliance with the American College of Cardiology/American Heart Association guidelines [1]. The modified Bruce protocol [22] was applied. 12-lead ECG, heart rate, and blood pressure were closely monitored, symptoms constantly evaluated, and parameters such as maximal rate pressure product and metabolic equivalents (METs) of estimated exercise capacity systematically reported [22,23].

EST was terminated by the physician in patients with: (a) maximal test, defined as an increase in heart rate either to ≥85% of the maximum predicted value off betablockers, or to ≥75% of the threshold on betablockers; EST was otherwise defined as submaximal; (b) sustained VT/VF, symptomatic NSVT, or significant increase in VE burden on effort, including polymorphic or bi/trigeminal beats; (c) angina and/or ST-T changes, including ST depression of ≥2 mm read at 60 to 80 ms from the J point, or new-onset negative T waves in at least two consecutive leads. EST was deemed uninterpretable for ischemia in the presence of baseline left bundle branch block.

For all VA documented on EST, 12-lead morphology and cycle length regularity were assessed as previously described [24].

### 2.5. Endpoints

The study endpoints, evaluated for both the study and control groups, included: (1) occurrence of VA during EST; (2) documentation of signs and/or symptoms of ischemia (diagnostic ST-T changes; angina-like chest pain with subsequent documentation of T-troponin raise) during EST; (3) occurrence of adverse events, namely cardiac death, disease-related hospital readmissions, malignant VA (sustained VT, VF, appropriate ICD treatment), and active myocarditis proven either by EMB, CMR, or FDG-PET after EST until the end of study (1 June 2022).

### 2.6. Statistical Analysis

SPSS Version 20 (IBM Corp., Armonk, NY, USA) was used for analysis and graphic presentations. Continuous variables were expressed as mean and standard deviation, or as median and interquartile range (IQR), depending on the distribution of data. Accordingly, they were compared by parametric (unpaired Student T) or non-parametric (Mann-Whitney U) tests, respectively. Survival curves were generated by the Kaplan-Meier method and compared by the log-rank test. Confidence intervals (CI) were set at 95%. Where relevant, 2-sided *p*-values < 0.05 were considered as statistically significant.

## 3. Results

### 3.1. Study Population

**Figure 1:**
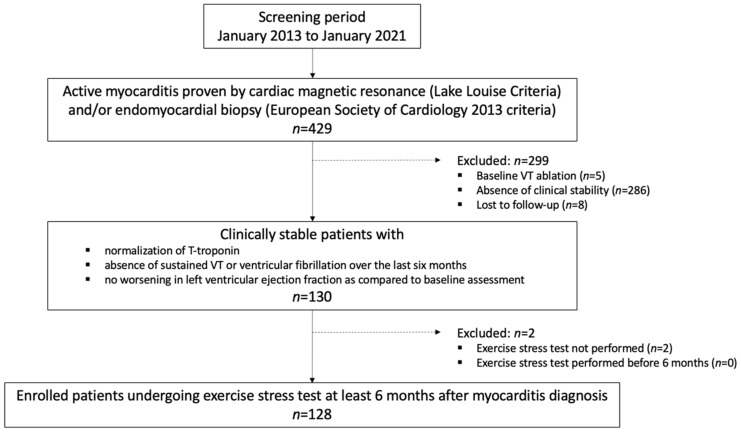
Patient selection. The study flowchart is shown to highlight the selection of the 128 patients undergoing an exercise stress test. VT = ventricular tachycardia.

The study population is composed of 128 consecutive patients (mean age 41 ± 9 years, males 70%) undergoing EST at least six months after myocarditis. Patient selection process and excluded cases are shown in Figure 1.

Of the 128 included patients, 64 (50%) presented with VA, namely VF/sustained VT in *n* = 32, NSVT in *n* = 18, and Lown’s grade ≥ 2 VE in *n* = 14 cases. Within the control group (*n* = 64), 34 (53%) had ACS-like clinical onset, while the remaining 30 (47%) presented with heart failure (HF). The cohort included up to 37 athletes (29%) who were previously eligible for agonistic sports practice. Overall, myocarditis was proven by CMR in 97 cases (76%, mainly nonarrhythmic), and by EMB in 116 (91%, mainly arrhythmic). Eighty-five patients (66%) had a diagnosis confirmed by both techniques. There were no cases of COVID-19-associated myocarditis. Patients were discharged from the hospital after 11 ± 4 days. Complete baseline characterization of the population and treatment strategies is shown in Table 1. Beta-blockers, antiarrhythmics, implantable cardioverter defibrillators, and immunosuppressants were all more commonly used in the arrhythmic group.

### 3.2. EST Results

**Figure 2:**
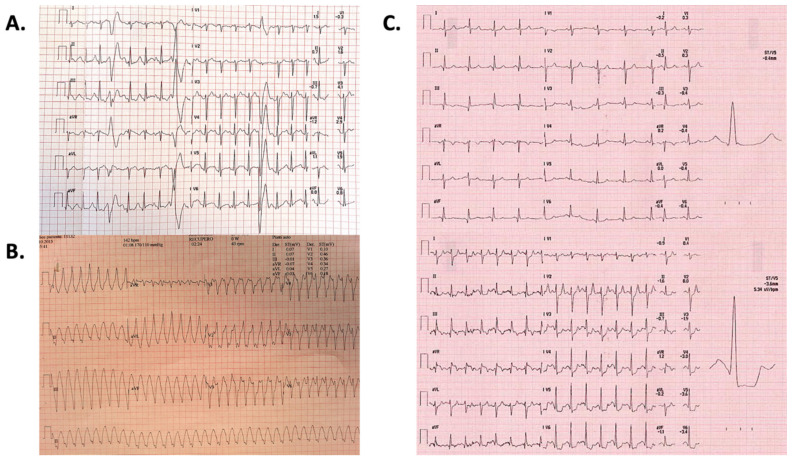
Exercise stress test findings. Representative examples of remarkable findings on exercise stress tests are shown. (Panel (**A**)). Female patient, 32-year-old, with evidence of frequent polymorphic ventricular ectopies on the effort at exercise stress test performed 12 months after cardiac magnetic resonance-proven acute myocarditis presenting with nonsustained ventricular tachycardia and syncope. After the exercise stress test, he underwent an endomyocardial biopsy and subsequent immunosuppressive treatment for virus-negative lymphocytic myocarditis. (Panel (**B**)). Male patient, 44-year-old (P123, Table 3) with evidence of sustained monomorphic ventricular tachycardia causing syncope during an exercise stress test performed late after presentation with arrhythmic myocarditis. The subsequent workup is shown in Table 3. (Panel (**C**)). Male patient, 68-year-old (P37, Table 3) with exercise stress test showing dynamic ST segment depression in inferolateral leads with ST elevation in lead aVR (exercise peak, bottom; compared to baseline, top). He was asymptomatic for angina or dyspnea. After normal coronary angiography, he had both cardiac magnetic resonance and endomyocardial biopsy with a final diagnosis of chronically active myocarditis from parvovirus B19. Further details are reported in Table 3.

Complete data about EST are summarized in Table 2. EST was performed on average after 15 ± 4 months from the diagnosis of myocarditis, with a later timing for the arrhythmic group. In addition, patients presenting with VA more commonly were tested on beta-blockers and/or antiarrhythmics (59 vs. 20, respectively, *p* < 0.001), accounting for lower average exercise performance. Overall, five patients (4%) experienced malignant VA, all of them belonging to the arrhythmic group. The same group showed a significantly higher occurrence of any kind of VA (43 vs. 4 cases, respectively, *p* < 0.001). Conversely, signs and/or symptoms of myocardial ischemia were more commonly documented in the nonarrhythmic group (6 vs. 1 cases, respectively, *p* = 0.115). Representative examples of EST findings are shown in Figure 2. Case-by-case management based on EST results is shown in Table 3.

### 3.3. Outcomes

**Figure 3:**
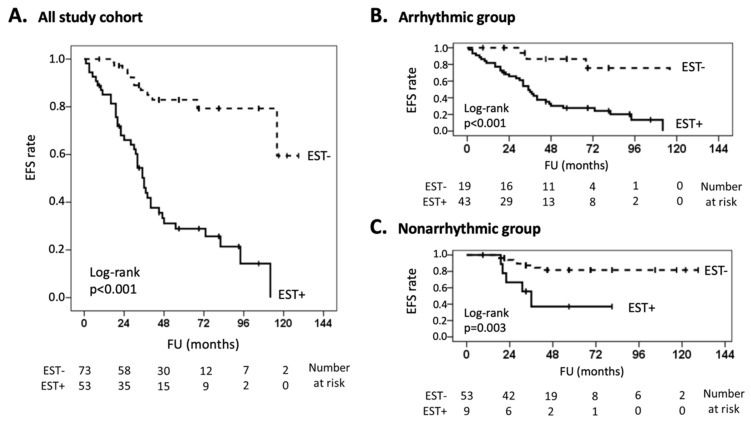
Outcomes after exercise stress test. Kaplan-Meier curves are shown for the occurrence of adverse events after EST. Adverse events included cardiac death, disease-related hospital readmissions, malignant ventricular arrhythmia (sustained ventricular tachycardia, ventricular fibrillation, appropriate implantable cardioverter defibrillator treatment), and active myocarditis, proven by endomyocardial biopsy or myocardial imaging. For each graph, *x*-axis indicates follow-up months after EST, and *y*-axis indicates event-free survival. The continuous line refers to patients with EST positive for ventricular arrhythmia or ischemia (EST+), whereas the dashed line refers to patients with uneventful EST (EST-). Numbers at risk are reported below each chart. Curves are shown for the whole patient cohort (panel (**A**)) and arrhythmic and nonarrhythmic subgroups (panels (**B**,**C**), respectively). EFS = event-free survival; EST = exercise stress test; FU = follow-up.

After average follow-up of 58 months, 52 patients (41%) experienced adverse events, including cardiac death (*n* = 3), rehospitalization (*n* = 37), malignant VA (*n* = 23) and proven myocarditis (*n* = 10). The global occurrence of adverse events was 39 (61%) in the arrhythmic vs. 13 (20%) in the nonarrhythmic group (*p* < 0.001). Event details are shown in Table 4. Remarkably, adverse events occurred more frequently among patients with abnormal EST findings (40/54 vs. 12/74, *p* < 0.001). As shown by the Kaplan-Meier curves in Figure 3, this difference was observed in both the arrhythmic and nonarrhythmic groups.

**Figure 4:**
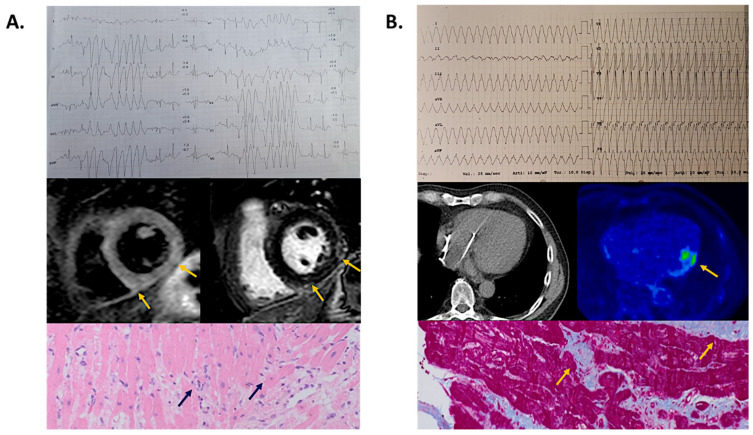
Relationship between exercise stress test-induced ventricular arrhythmia and myocarditis staging. Representative examples of ventricular arrhythmia features on exercise stress test and subsequent myocarditis restaging are shown. (Panel (**A**)). Female patient, 40-year-old, with evidence of irregular nonsustained ventricular tachycardia immediately after exercise peak on stress test. She underwent cardiac magnetic resonance showing both T2 short-tau inversion recovery (mid panel, left) and late gadolinium enhancement sequences (mid panel, right) involving the subepicardial layer of the inferolateral left ventricular wall (arrows), fulfilling the Lake Louise criteria for active myocarditis. She underwent an endomyocardial biopsy showing multifocal lymphocytic inflammatory infiltrates (lower panel, arrows) confirming the diagnosis of chronically active virus-negative myocarditis, subsequently treated by immunosuppressants. (Panel (**B**)). Male patient, 66-year-old (P78, Table 3) with evidence of regular and monomorphic sustained ventricular tachycardia causing syncope during an exercise stress test performed late after presentation with arrhythmic myocarditis. Being an implantable cardioverter defibrillator carrier (mid panel, left), he underwent ^18^F-Fluorodeoxyglucose positron emission tomography (mid panel, right) with physiological glucose uptake in the mid-lateral left ventricular wall (arrow) and no signs of active myocarditis. Consistently, endomyocardial biopsy (lower panel) showed only replacement fibrosis (arrows) and no signs of persistent myocardial inflammation. He successfully underwent catheter ablation of monomorphic ventricular tachycardia (Table 3).

Of 47 patients with VA documented during EST, 35 (74%) underwent myocarditis restaging either by CMR (*n* = 19), FDG-PET (*n* = 15), or EMB (*n* = 8): prevalence of polymorphic and/or irregular VA during EST were observed in patients with subsequent documentation of active myocarditis (6 of 7), whereas regular and monomorphic VA were more common among patients with no signs of active myocarditis (26 of 28, *p* < 0.001). Examples are shown in Figure 4.

Among the 33 patients with uneventful EST on treatment, 16 (48%) had beta-blocker withdrawn (2/14 arrhythmic vs. 14/19 nonarrhythmic, *p* = 0.001) and subsequently underwent EST under off-treatment conditions: there were no cases of inducible ischemia and no major VA. Only two patients, those belonging to the arrhythmic group, required beta-blocker resumption, respectively, because of NSVT and frequent VE. Of the 27 athletes of the cohort, 12 cases (44%), all belonging to the nonarrhythmic group, were readmitted to competitive sports participation.

## 4. Discussion

### 4.1. Main Study Findings

We reported the results of EST late after myocarditis in a sizable cohort of patients with a balanced distribution between arrhythmic and nonarrhythmic presentations. We showed that: (1) the occurrence of VA during EST was more common in the arrhythmic presentation, whereas ischemic manifestations were more prevalent in the nonarrhythmic one; (2) in both arrhythmic and nonarrhythmic groups, adverse events occurred more frequently among patients with abnormal EST; (3) EST was an informative technique for the subsequent clinical management of patients with myocarditis.

### 4.2. EST after Myocarditis: Role of the Clinical Presentation

To the best of our knowledge, we provided the first report comparing the results of EST in patients with arrhythmic and nonarrhythmic myocarditis. Myocarditis was diagnosed by gold standard techniques [2,13,14] and followed at a dedicated outpatient clinic with multidisciplinary facilities [20]. Furthermore, in compliance with the current recommendations [4,5], EST was performed at least six months after acute myocarditis and provided a clinical judgment of stability for at least six months. In keeping with the current knowledge, EST was safe in the nonarrhythmic cohort, where malignant VA was never documented. As opposed, we documented a high prevalence of VA, including five cases of malignant ones, among patients with an arrhythmic presentation. Results are not unexpected, since VA may complicate both the active and the post-inflammatory stages of myocarditis [10,24]. Furthermore, evidence suggests that presentation with malignant VA predicts the subsequent occurrence of arrhythmic events in patients with myocarditis [25]. Our findings suggest that in patients with an arrhythmic presentation of myocarditis, EST should be requested with caution. Regarding inducible ischemia, we identified no patients with detectable abnormalities in epicardial coronary arteries (Table 3). Due to the subsequent diagnosis of chronically active myocarditis in most cases, the observed ST-T changes likely occurred secondary to structural heart disease-associated coronary microvascular dysfunction [26], as already demonstrated in patients with myocarditis secondary to viruses with endothelial tropism like parvovirus B19 [27].

### 4.3. Significance of EST

Figure 3 shows that the vast majority of adverse events during follow-up occurred in patients with abnormal findings at EST. To be noted, a number of known prognostic factors in patients with myocarditis, including male gender [28], LVEF [9], LGE [25], viral genomes [29], and wide QRS complex from left bundle branch block [30] displayed a balanced distribution between groups (Table 1). Also, relevant comorbidities [31,32,33] showed no major differences in arrhythmic vs. nonarrhythmic cases. However, a major difference between groups was found in medical treatment. In particular, beta-blockers and antiarrhythmic agents were largely more prevalent among the arrhythmic patients, who nevertheless experienced a greater rate of adverse events (Table 4).

Table 4 shows that the majority of adverse events occurred in patients with abnormal EST. However, multiple factors might have contributed to our findings. First, the majority of adverse events occurred in the arrhythmic group, which is already known for being associated with worse outcomes [6,7,25]. Second, a non-trivial subset of patients with abnormal EST (7 of 35, 20%) received a diagnosis of active myocarditis following the subsequent disease restaging: this observation suggests that adverse prognostic significance may be carried out by active myocardial inflammation, as previously reported [34]. Beyond any prognostic significance, EST remains an informative technique in daily clinical practice since the new documentation of VA may help identify high-risk patients who warrant treatment upgrade and close follow-up.

### 4.4. Additional Clinical Implications of EST

Our study was notable for a number of additional findings that may guide the clinical decision-making in patients undergoing EST late after myocarditis. First, the features of VA at 12-lead ECG may be a useful tool to identify the inflammatory stage of myocarditis. Previously, polymorphic and irregular VA was shown to be associated with active-phase myocarditis, whereas monomorphic and regular ones suggested post-inflammatory scar-related VA [24]. This distinction has been proven to turn into relevant differences in treatment strategies, ranging from immunosuppression for the former condition [35] and catheter ablation for the latter one [34]. Overall, the results of the current study are in keeping with this observation (Figure 4) and suggest that persistently active myocarditis could be suspected even in clinically-stable patients who show polymorphic and irregular VA on EST performed late after the acute presentation.

A second relevant point is that a normal EST may guide the withdrawal of medical treatment. In our experience, all nonischemic patients had uneventful EST even later, under off-treatment conditions. Most importantly, all of them had no adverse events during follow-up. These data suggest that, at least in patients with nonarrhythmic presentation and no alternative indications (i.e., heart failure), beta-blockers may be safely interrupted after uneventful EST. These findings are relevant, in particular for young patients requesting readmission to competitive sports participation [4,5], who constituted a non-neglectable subset of our cohort (29%). As opposed, an arrhythmic presentation may constitute a limitation for the subsequent readmission to sports practice. Our data are meant to be preliminary and deserve confirmation by larger, multicenter studies.

### 4.5. Study Limitations

Our study was single-center and took place at a referral center for arrhythmic myocarditis [18]. This may have led to an overestimation of the prevalence of VA as compared to the classic ACS-like and HF presentations of myocarditis [3]. Considerable overlap between acute and chronically active myocarditis, as well as improved diagnostic yield of modern CMR following the introduction of parametric mapping, constitute additional biases. Continuous electrical monitoring by cardiac devices in many patients allowed greater sensitivity for arrhythmia detection as compared with the classic approach based on repeated Holter ECG [36,37]. Due to the limited sample size, the statistical model allowing assessment of the independent prognostic value for EST was prevented.

## 5. Conclusions

In this study, we showed that EST, performed late after the clinical onset of myocarditis, is more commonly associated with VA in patients with arrhythmic presentation compared to nonarrhythmic ones. Furthermore, we showed that EST abnormalities are associated with adverse outcomes during subsequent follow-ups in both arrhythmic and nonarrhythmic groups. These preliminary findings suggest that EST should be requested and performed with caution in patients with myocarditis, in particular following the arrhythmic presentation. Confirmatory evidence by larger studies is needed.

## Figures and Tables

**Table 1:** Baseline characteristics of population (*n* = 128).

		Arrhythmic *n* = 64	Nonarrhythmic *n* = 64	*p*
Clinical features				
Age (y) Male gender	Mean ± SD *n* (%)	42 ± 10 45 (70)	40 ± 9 44 (68)	0.237 1.000
History of myocarditis History of SCD or CMP Agonism	*n* (%) *n* (%) *n* (%)	3 (5) 5 (8) 17 (27)	4 (6) 4 (6) 20 (31)	1.000 1.000 0.697
Anemia Thyroid dysfunction SIDs	*n* (%) *n* (%) *n* (%)	7 (11) 9 (14) 6 (9)	6 (9) 7 (11) 4 (6)	1.000 0.790 0.744
Presentation				
ACS-like HF Sustained VT/VF NSVT VE Lown’s grade ≥ 2 *	*n* (%) *n* (%) *n* (%) *n* (%) *n* (%)	0 (0) 0 (0) 32 (50) 18 (28) 14 (22)	34 (53) 30 (47) 0 (0) 0 (0) 0 (0)	<0.001 <0.001 <0.001 <0.001 <0.001
Blood exams				
T-Troponin (ng/L) NTproBNP (pg/mL) C-reactive protein (mg/L)	Median ± IQR Median ± IQR Median ± IQR	46 (19–312) 507 (118–1965) 5 (3–14)	78 (22–517) 396 (89–2170) 6 (3–25)	0.326 0.512 0.618
ECG				
PQ (ms) QRS (ms) QTc (ms) LBBB	Mean ± SD Mean ± SD Mean ± SD *n* (%)	174 ± 39 103 ± 24 416 ± 31 3 (5)	168 ± 42 99 ± 26 409 ± 33 5 (8)	0.404 0.368 0.218 0.718
Echocardiogram				
LVEDVi (mL/m^2^) LVEF (%) E/E’ RVEDD (mm) TAPSE (mm) Pericardial effusion	Mean ± SD Mean ± SD Mean ± SD Mean ± SD Mean ± SD *n* (%)	72 ± 20 50 ± 10 7 ± 2 29 ± 3 21 ± 3 2 (3)	68 ± 28 52 ± 16 7 ± 3 29 ± 4 22 ± 4 6 (9)	0.404 0.398 1.000 1.000 0.312 0.273
Myocarditis diagnosis				
CMR-proven (LLC) STIR, T2 LGE, T1, ECV EMB-proven (ESC criteria) CD3+ TCL > 7/mm^2^ Viral PCR	*n* (%) *n* (%) *n* (%) *n* (%) *n* (%) *n* (%)	39 (61) 39 (61) 60 (94) 62 (97) 62 (97) 7 (11)	58 (91) 58 (91) 60 (94) 54 (84) 54 (84) 13 (20)	<0.001 <0.001 1.000 0.030 0.030 0.223
Treatment at discharge				
ACE-inhibitors Betablockers Diuretics Antiarrhythmics IST ICD	*n* (%) *n* (%) *n* (%) *n* (%) *n* (%) *n* (%)	56 (88) 61 (95) 7 (11) 50 (78) 49 (77) 30 (47)	50 (78) 47 (73) 14 (22) 3 (5) 37 (58) 9 (14)	0.241 0.001 0.151 <0.001 0.038 <0.001

Baseline clinical features of the population (*n* = 128) and comparison between arrhythmic and nonarrhythmic groups are shown. * Lown’s grade ≥ 2 indicates > 1 VE per min, or > 30 VE per h. ACE = angiotensin converting enzyme; ACS = acute coronary syndrome; CD = cluster of differentiation; CMP = cardiomyopathy; CMR = cardiac magnetic resonance; ECV = extracellular volume; EMB = endomyocardial biopsy; ESC = European Society of Cardiology; HF = heart failure; ICD = implantable cardioverter defibrillator; IST = immunosuppressive therapy; LBBB = left bundle branch block; LGE = late gadolinium enhancement; LLC = Lake Louise criteria; LVEDVi = left ventricular end-diastolic volume (indexed); LVEF = left ventricular ejection fraction; NSVT = nonsustained ventricular tachycardia; PCR = polymerase chain reaction; RVEDD = right ventricular end-diastolic diameter (RV2); SCD = sudden cardiac death; SD = standard deviation; SIDs = systemic immune diseases; STIR = short-tau inversion recovery; TAPSE = tricuspid annulus plane systolic excursion; TCL = T-cell lymphocytes; VA = ventricular arrhythmia; VE = ventricular ectopies; VF = ventricular fibrillation; VT = ventricular tachycardia.

**Table 2:** Exercise stress test.

		Total *n* = 128	Arrhythmic *n* = 64	Nonarrhythmic *n* = 64	*p*
Time from clinical presentation	Mean ± SD	15 ± 4	19 ± 4	12 ± 3	<0.001
Treadmill Bicycle	*n* (%) *n* (%)	117 (91) 11 (9)	59 (92) 5 (8)	58 (91) 6 (9)	1.000 1.000
On treatment on betablockers on antiarrhythmics Off treatment	*n* (%) *n* (%) *n* (%) *n* (%)	79 (62) 75 (59) 14 (11) 49 (38)	59 (92) 55 (86) 14 (22) 5 (8)	20 (31) 20 (31) 0 (0) 44 (69)	<0.001 <0.001 <0.001 <0.001
Maximal power (W) Maximal METs Peak SBP (mmHg) Peak HR (bpm) Peak RPP (*10^2^) % MTHR (%)	Mean ± SD Mean ± SD Mean ± SD Mean ± SD Mean ± SD Mean ± SD	142 ± 9 10 ± 3 157 ± 14 150 ± 12 24 ± 5 85 ± 6	133 ± 8 9 ± 2 153 ± 16 146 ± 13 23 ± 5 84 ± 6	151 ± 11 11 ± 4 159 ± 15 154 ± 14 25 ± 6 86 ± 6	<0.001 0.001 0.031 0.001 0.043 0.062
Maximal negative test on betablockers off betablockers Submaximal negative test on betablockers off betablockers Positive test on betablockers off betablockers	*n* (%) *n* (%) *n* (%) *n* (%) *n* (%) *n* (%) *n* (%) *n* (%) *n* (%)	64 (50) 24 (19) 40 (31) 10 (8) 9 (7) 1 (1) 54 (42) 42 (33) 12 (9)	14 (22) 8 (13) 6 (9) 6 (9) 6 (9) 0 (0) 44 (69) 41 (64) 3 (5)	50 (78) 16 (25) 34 (53) 4 (6) 3 (5) 1 (2) 10 (16) 1 (2) 9 (14)	<0.001 0.112 <0.001 0.744 0.492 1.000 <0.001 <0.001 0.127
VA Sustained VT/VF * NSVT VE	*n* (%) *n* (%) *n* (%) *n* (%)	47 (37) 5 (4) 24 (19) 40 (31)	43 (67) 5 (8) 24 (38) 36 (56)	4 (6) 0 (0) 0 (0) 4 (6)	<0.001 0.058 <0.001 <0.001
Ischemia * ST-T changes Angina-like chest pain Uninterpretable for LBBB	*n* (%) *n* (%) *n* (%) *n* (%)	7 (5) 4 (3) 3 (2) 8 (6)	1 (2) 0 (0) 1 (2) 3 (5)	6 (9) 4 (6) 2 (3) 5 (8)	0.115 0.119 1.000 0.718

Results of the exercise stress test are shown for the whole cohort and study groups. * Detail about baseline features and subsequent management of patients with sustained VT/VF or evidence of ischemia during exercise stress tests are shown in Table 3. HR = heart rate; METs = metabolic equivalents; MPHR = maximal predicted heart rate; NSVT = nonsustained ventricular tachycardia; RPP = rate pressure product; SBP = systolic blood pressure; VA = ventricular arrhythmia; VE = ventricular ectopies; VF = ventricular fibrillation; VT = ventricular tachycardia.

**Table 3:** Management of patients with ischemia and malignant VA during exercise stress test.

PID	Age (y)	Gender	Presentation	Baseline LVEF (%)	Baseline Myocarditis	Baseline Treatment	Malignant VA during EST	Management
P45	54	Male	NSVT	55	EMB-proven, virus-negative	sotalol, ramipril	Presyncopal sustained VT	ICD implant. EMB: chronically active virus-negative lymphocytic myocarditis. IST for 12 months until FDG-PET normalization
P64	32	Male	Sustained VT	60	CMR-proven	metoprolol	Tolerated sustained VT	EMB: chronically active virus-negative lymphocytic myocarditis. IST for 12 months until CMR normalization. Flecainide
P67	34	Male	VF	51	EMB-proven, viral	metoprolol, amiodarone, ICD	Presyncopal sustained VT	VT ablation. Subsequent uneventful follow-up
P78	34	Male	Sustained VT	66	EMB-proven, virus-negative	flecainide, metoprolol, prior IST (prednisone, azathioprine), ICD	Tolerated sustained VT	FDG-PET: normal. EMB: replacement fibrosis, no myocarditis. VT ablation. Subsequent uneventful follow-up
P124	44	Male	Sustained VT	60	EMB-proven, virus-negative	flecainide, metoprolol, prior IST (prednisone, azathioprine), ICD	Syncopal sustained VT	EMB: replacement fibrosis, no myocarditis. VT ablation. Subsequent uneventful follow-up
**PID**	**Age** **(y)**	**Gender**	**Presentation**	**Baseline LVEF (%)**	**Baseline** **Myocarditis**	**Baseline** **Treatment**	**Ischemia** **during EST**	**Management**
P37	68	Male	ACS-like	58	CMR-proven	ramipril, ivabradine	ST-T changes, asymptomatic	Coronary angiography: normal. CMR: persistently active myocarditis. EMB: chronically active viral lymphocytic myocarditis (parvovirus B19). No etiology-specific treatment
P41	59	Male	ACS-like	60	CMR-proven and EMB-proven, virus-negative	ramipril, prior IST (prednisone, azathioprine)	Angina-like chest pain, no ST-T changes	Coronary CT scan: normal. CMR: persistently active myocarditis. EMB: chronically active virus-negative lymphocytic myocarditis. IST for additional 6 months until CMR normalization
P63	41	Male	HF	25	CMR-proven after LVEF recovery up to 50%	enalapril, furosemide	ST-T changes, asymptomatic	Coronary CT scan: normal. CMR: normal. Bisoprolol. No additional diagnostic workup
P98	40	Male	HF	38	CMR-proven	sacubitril/valsartan	ST-T changes, asymptomatic	Coronary CT scan: normal. CMR: persistently active myocarditis. EMB: chronically active virus-negative lymphocytic myocarditis. Bisoprolol and IST for 12 months until CMR normalization
P99	52	Male	ACS-like	55	EMB-proven, virus-negative	ramipril, prior IST (prednisone, azathioprine)	Angina-like chest pain, no ST-T changes, abnormal T-troponin	Coronary CT scan: normal. CMR: persistently active myocarditis. EMB: chronically active virus-negative lymphocytic myocarditis. Bisoprolol and IST for additional 6 months until CMR normalization
P103	64	Male	ACS-like	62	CMR-proven	none	Angina-like chest pain, no ST-T changes	CMR: normal. Coronary CT scan: normal. No additional diagnostic workup
P119	23	Female	Sustained VT	44	CMR-proven and EMB-proven, virus-negative	metoprolol, ramipril, prior IST (prednisone, azathioprine), ICD	Angina-like chest pain, no ST-T changes, abnormal T-troponin	FDG-PET scan: persistently active myocarditis. EMB: chronically active virus-negative lymphocytic myocarditis. IST for additional 12 months until FDG-PET normalization

Details about patients with sustained VT/VF (*n* = 5) or evidence of ischemia during EST (*n* = 7) are shown, together with the subsequent case by case management. ACS = acute coronary syndrome; CT = computed tomography; EMB = endomyocardial biopsy; EST = exercise stress test; FDG-PET = ^18^F-Fluorodeoxyglucose positron emission tomography; HF = heart failure; ICD = implantable cardioverter defibrillator; IST = immunosuppressive therapy; LVEF = left ventricular ejection fraction; NSVT = nonsustained ventricular tachycardia; VA = ventricular arrhythmia; VF = ventricular fibrillation; VT = ventricular tachycardia.

**Table 4:** Outcomes after exercise stress test.

		Total *n* = 128	Arrhythmic *n* = 64	Nonarrhythmic *n* = 64	*p*	EST+ *n* = 54	EST- *n* = 74	*p*
Adverse events	*n* (%)	52 (41)	39 (61)	13 (20)	<0.001	40 (74)	12 (16)	<0.001
Cardiac death	*n* (%)	3 (2)	3 (5)	0 (0)	0.244	3 (6)	0 (0)	0.073
Disease-related hospitalizations	*n* (%)	37 (29)	24 (38)	13 (20)	0.050	27 (50)	10 (14)	<0.001
Malignant VA *	*n* (%)	23 (18)	21 (33)	2 (3)	<0.001	19 (35)	4 (5)	<0.001
Proven active myocarditis	*n* (%)	10 (8)	7 (11)	3 (5)	0.324	8 (15)	2 (3)	0.017

Outcomes of the whole cohort and study groups are shown. * Malignant VA include sustained ventricular tachycardia, ventricular fibrillation, and appropriate implantable cardioverter defibrillator therapy. EST = exercise stress test; VA = ventricular arrhythmia.

## Data Availability

The data supporting the study findings will be made available upon reasonable request.

## Abbreviations

ACS = acute coronary syndrome; CMR = cardiac magnetic resonance; ECG = electrocardiogram; EMB = endomyocardial biopsy; EST = exercise stress test; FDG-PET = 18F-Fluorodeoxyglucose positron emission tomography; HF = heart failure; ICD = implantable cardioverter defibrillator; IST = immunosuppressive therapy; LGE = late gadolinium enhancement; LLC = Lake Louise criteria; LVEF = left ventricular ejection fraction; NSVT = nonsustained ventricular tachycardia; VA = ventricular arrhythmia; VE = ventricular ectopies; VF = ventricular fibrillation; VT = ventricular tachycardia.
